# bFGF blockade reduces intraplaque angiogenesis and macrophage infiltration in atherosclerotic vein graft lesions in ApoE3*Leiden mice

**DOI:** 10.1038/s41598-020-72992-7

**Published:** 2020-09-29

**Authors:** Laura Parma, Hendrika A. B. Peters, Thijs J. Sluiter, Karin H. Simons, Paolo Lazzari, Margreet R. de Vries, Paul H. A. Quax

**Affiliations:** 1 Department of Vascular Surgery, D6-33, Leiden University Medical Center, PO Box 9600, 2300 RC Leiden, The Netherlands; 2 Einthoven Laboratory for Experimental Vascular Medicine, Leiden University Medical Center, Leiden, The Netherlands; 3 KemoTech SrL, Build 3, Loc. Piscinamanna, 09010 Pula, Italy

**Keywords:** Cardiovascular biology, Atherosclerosis

## Abstract

Intraplaque angiogenesis increases the chance of unstable atherosclerotic plaque rupture and thrombus formation leading to myocardial infarction. Basic Fibroblast Growth Factor (bFGF) plays a key role in angiogenesis and inflammation and is involved in the pathogenesis of atherosclerosis. Therefore, we aim to test K5, a small molecule bFGF-inhibitor, on remodelling of accelerated atherosclerotic vein grafts lesions in ApoE3*Leiden mice. K5-mediated bFGF-signalling blockade strongly decreased intraplaque angiogenesis and intraplaque hemorrhage. Moreover, it reduced macrophage infiltration in the lesions by modulating CCL2 and VCAM1 expression. Therefore, K5 increases plaque stability. To study the isolated effect of K5 on angiogenesis and SMCs-mediated intimal hyperplasia formation, we used an in vivo Matrigel-plug mouse model that reveals the effects on in vivo angiogenesis and femoral artery cuff model to exclusively looks at SMCs. K5 drastically reduced in vivo angiogenesis in the matrigel plug model while no effect on SMCs migration nor proliferation could be seen in the femoral artery cuff model. Moreover, in vitro K5 impaired endothelial cells functions, decreasing migration, proliferation and tube formation. Our data show that K5-mediated bFGF signalling blockade in hypercholesterolemic ApoE3*Leiden mice reduces intraplaque angiogenesis, haemorrhage and inflammation. Therefore, K5 is a promising candidate to stabilize advanced atherosclerotic plaques.

## Introduction

Atherosclerosis is a chronic disease that culminates with plaque rupture often leading to acute cardiovascular events. Crucial factors for plaque instability and therefore for plaque rupture are not the size, but rather the composition of the atherosclerotic plaques^1^. Distinctive features like large necrotic cores, intraplaque angiogenesis, intraplaque haemorrhage, high macrophage content and a thin fibrous cap are known for their role in plaque destabilization^2^.

Basic fibroblast growth factor (bFGF) is involved in the pathogenesis of atherosclerosis^3^ and especially in the regulation of processes that drive plaque instability^4,5^. It is a known regulator of angiogenesis, macrophage infiltration as well as smooth muscle cells (SMCs) fate^6,7^. A member of the fibroblast growth factors family, bFGF is secreted by SMCs and macrophages^8,9^ and exerts its activities by binding to one of the four FGF receptors (FGFR1-4) on the cell surface of different cell types, among which endothelial cells (ECs) and SMCs. bFGF exploits its function on ECs mostly via binding to FGFR1^10,11^. Upon bFGF binding the receptor dimerizes, gets phosphorylated and the complex ligand/receptor is internalized, resulting in signalling for cell motility, proliferation and survival, leading to angiogenesis^12^.

In atherosclerotic lesions, bFGF was shown to promote intraplaque angiogenesis^13^. Intraplaque angiogenesis is driven by local hypoxia that triggers the formation of neovessels from the vasa vasora in the adventitia^14,15^. These newly formed neovessels are immature and leaky. They lack a continuous pericyte coverage and regular tight junctions between endothelial cells, leading to intraplaque haemorrhage. Intraplaque haemorrhage, the extravasation of red blood cells into the plaque, along with inflammatory cells fuels the ongoing inflammation. At the same time, the invading inflammatory cells and in particular macrophages promote the synthesis of various angiogenic factors^16,17^, further triggering plaque angiogenesis^15^. bFGF also enhances the infiltration of macrophages in the lesions via stimulating the expression of chemokines and adhesion molecules like MCP-1 and VCAM-1^6^. Accumulation of (macrophage derived) foam cells contributes to lipid storage, atherosclerotic plaque growth and drives the plaque pro-inflammatory phenotype^18^. As a result, intraplaque angiogenesis and inflammation are strongly connected in atherosclerosis and form a vicious cycle that drives the atherosclerotic plaque toward an unstable phenotype and possibly rupture.

For many years bFGF has been known to promote SMC proliferation and migration^19^ and it was a major target in the prevention of post-interventional intimal hyperplasia^3,20^. SMCs play a complex role in the stability of atherosclerotic lesions. They are involved in lesion formation by inducing intimal proliferation, but are also crucial in the formation of the fibrous cap covering the lesion. Upon vascular injury or endothelial cell activation SMCs display a switch from a contractile phenotype towards a proliferative phenotype characterized by enhanced cell proliferation and migration^21^. SMCs in the fibrous cap mainly show a contractile phenotype^22^ and the thickness of the cap is crucial for plaque stability since plaque rupture is increased by cap thinning^23^. bFGF was found to be higher expressed in SMCs of unstable plaques when compared to stable plaques^5^ and is known to stimulate a proliferative phenotype^19^.

We hypothesize that blocking bFGF signalling would potentially reduce intraplaque angiogenesis, macrophage infiltration and SMCs proliferation, resulting in stabilization of atherosclerotic plaques.

**Figure 1:**
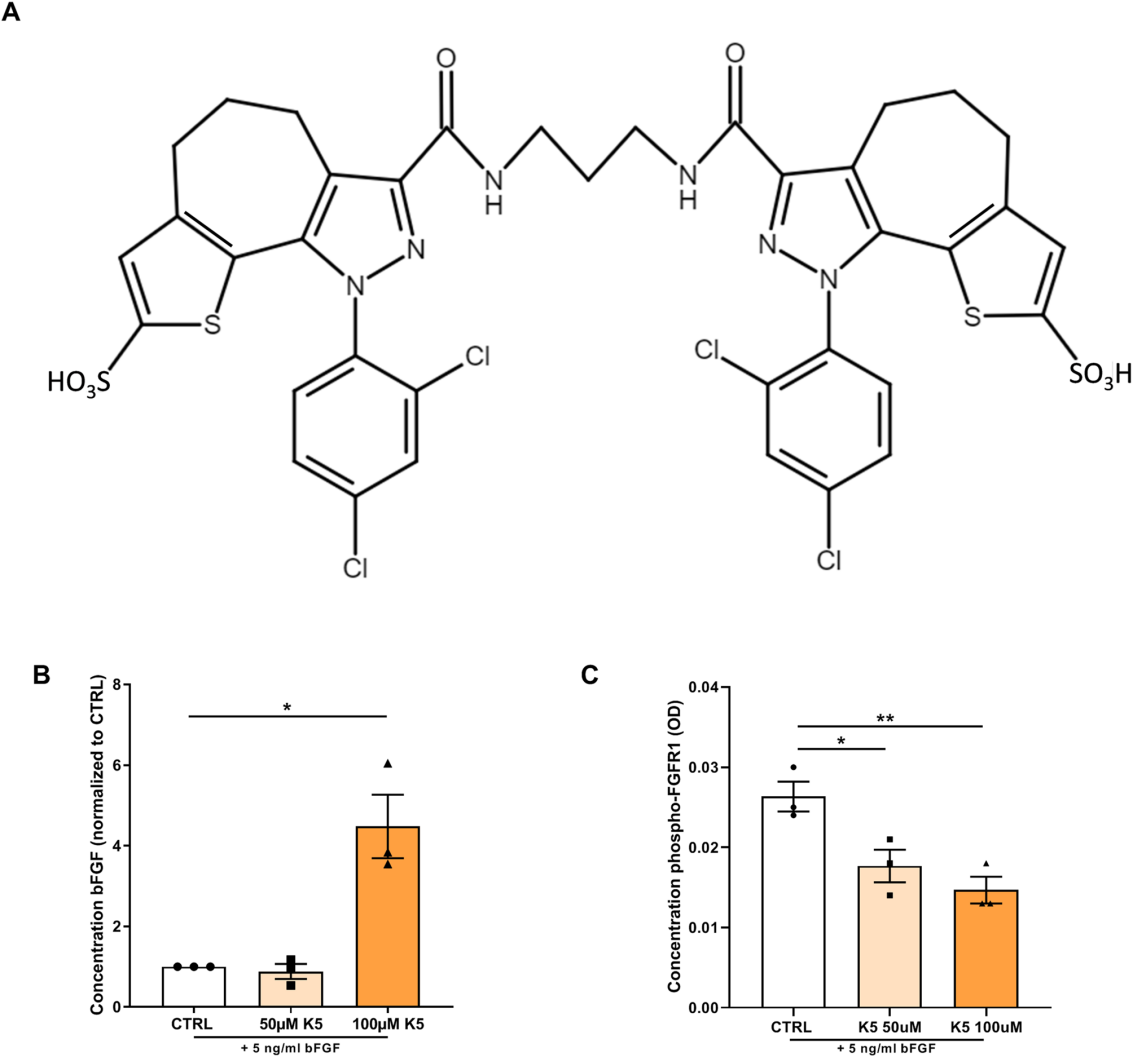
K5 prevents bFGF internalization and inhibits FGFR1 phosphorylation. (**A**) Chemical structure of K5. (**B**) Quantification of the concentration of bFGF in cell culture medium of H5V cells incubated with increasing doses of K5 and 5 ng/ml of bFGF (n = 3 technical replicates). (**C**) Concentration of phosphorylated FGFR1 in HUVECs treated with two different concentration of K5 (n = 3 technical replicates). Data are presented as mean ± SEM. *p < 0.05, **p < 0.01, ***p < 0.001. ****p < 0.0001; by 2-sided Student t test.

3,3′-(propane-1,3-diyilbis(azanediyl)bis(oxomethylene)bis(1-(2,4-dichlorophenyl)-1,4-dihydro-thieno[3′,2′:4,5]cyclohepta[1,2-c]pyrazole-8-sulfonic acid), namely K5 (Fig. 1) a small molecule developed by Kemotech Srl (Parco Scientifico di Pula, Cagliari, Italy), is able to bind bFGF and act as inhibitor of bFGF signalling^24^.

The effect of K5-mediated bFGF signalling blockade on intraplaque angiogenesis, inflammation and SMCs proliferation in atherosclerosis was studied in accelerated atherosclerotic lesions vein graft in hypercholesterolemic ApoE3*Leiden mice. We previously showed that such lesions deeply resemble atherosclerotic unstable plaques found in humans, containing hypoxia, intraplaque angiogenesis and haemorrhage, profound intimal hyperplasia with a strong SMCs proliferation component and extensive inflammation^25^. In this murine model intraplaque angiogenesis can be detected as early as 14 days and after 28 days neovessels can be found throughout all layers of the vein graft. These neovessels have either a basement membrane and a pericyte coverage^26^, characteristics of mature plaque neovessels, or they lack a complete pericyte coverage, peculiarity of immature neovessels, both found in native atherosclerosis. These characteristics allow for the study of both intraplaque angiogenesis and intraplaque haemorrhage in this murine model.

To study the isolated effect of K5 on angiogenesis and SMC proliferation, we evaluated also the effects of K5 in vivo in a matrigel plug mouse model that exclusively reveals the effects on in vivo angiogenesis and on in vivo femoral artery cuff model that looks solely at the effects of SMCs mediated intimal hyperplasia formation. Moreover, we investigated the effect of K5 on cultured endothelial cells using in vitro angiogenesis models.

Hence, we hypothesized that K5 treatment will reduce intraplaque angiogenesis, inflammation and SMCs proliferation in vein graft lesions in hypercholesterolemic ApoE3*Leiden mice and therefore increase lesion stability.

## Results

### K5 prevents bFGF internalization and inhibits FGFR1 phosphorylation

We first analysed whether K5 (chemical structure in Fig. 1A) prevents the receptor binding and internalisation of bFGF by measuring the levels of bFGF in the culture medium of H5V endothelial cells treated with K5 and comparing it to untreated cells. In the conditioned medium of K5 treated cells we observed a significant accumulation of bFGF, that reached with the highest dose tested of 100 µM a four-fold difference when compared to control (Fig. 1B, p = 0.01).

Moreover, in order to demonstrate that K5 treatment resulted in reduced activation and therefore reduced phosphorylation of FGFR1, we quantified the degree of phosphorylation of FGFR1 in HUVECs treated with K5 or control treatment. As shown in Fig. 1C, both doses tested resulted in a significant dose dependent (*p* = 0.03 and 0.009 respectively) reduction, by 35% and 46%, in phosphorylation of the receptor compared to the control group.

### Effects of K5 on vessel wall remodelling and lesion composition in vein grafts

We studied the effect of K5 on vessel wall remodelling in an in vivo model of accelerated atherosclerosis in hypercholesterolemic ApoE3*Leiden mice which underwent vein graft surgery.

**Figure 2:**
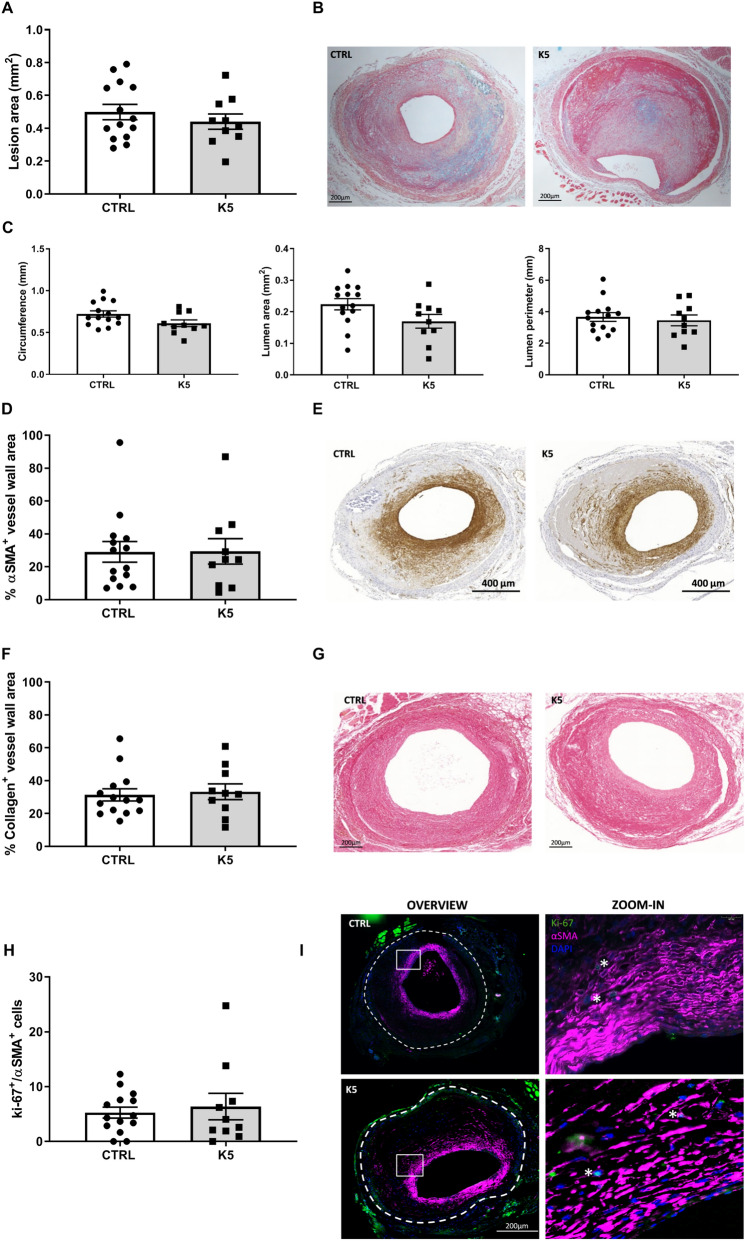
K5 does not affect neointima formation nor SMCs content in advanced vein graft atherosclerotic plaques in ApoE3*Leiden mice. (**A**) Quantification of lesion area 28 days after vein graft surgery in ApoE3*Leiden mice and (**B**) representative pictures of the lesions of control and K5 treated mice (n = 14 in CTRL group and n = 10 in K5 group). (**C**) Quantification of lesion circumference, lumen area and perimeter (n = 14 in CTRL group and n = 10 in K5 group). (**D**) Quantification of αSMA positive area in the vessel wall and (**E**) representative examples of CTRL and K5 groups lesions (n = 14 in CTRL group and n = 10 in K5 group). (**F**) Quantification of sirius red positive lesion area and (**G**) examples of the staining in vein graft of CTRL and K5 treated groups (n = 14 in CTRL group and n = 10 in K5 group). (**H**) Quantification of Ki-67 and αSMA double positive cells and (I) examples of immunofluorescent staining for CTRL and K5 treated groups (n = 14 in CTRL group and n = 10 in K5 group). The white dotted line delineates the perimeter of the lesion. Data are presented as mean ± SEM *p < 0.05, **p < 0.01, ***p < 0.001. ****p < 0.0001; by 2-sided Student t test.

Treatment with K5 at the dose of 25 mg/kg was well tolerated by the mice and did not affect weight nor cholesterol levels (Supplementary information 1). Mice treated with K5 (n = 10) had comparable vessel wall thickening to mice of the vehicle treated control group (n = 14) (Fig. 2A,B. *p* = 0.4) . Moreover, when looking in detail at the vascular remodelling in the lesions, no differences in circumference of the vessel, lumen area or lumen perimeter could be detected in the K5 treated mice when compared to controls (Fig. 2C).

bFGF is known to regulate SMCs migration and proliferation and inhibition or lack of these processes are crucial drivers toward the formation of advanced unstable atherosclerotic lesions. To assess the effect of K5 on SMCs content in atherosclerotic lesions, we performed an anti-αSMA immunohistochemical staining. αSMA^+^ cells were found, both in the K5 treated and in the control groups, mainly in the intimal layer of the vessel around the lumen (Fig. 2E). When quantified, the total amount of SMCs in the K5 treated group showed no differences compared to control (Fig. 2D. *p* = 0.97), not was the number of proliferating SMCs (Fig. 2I) different between the lesions of K5 treated and control mice (Fig. 2H).

Since SMCs are the main producers of collagen, we analysed the collagen content in the lesions. Sirius red positive areas were detected in both groups and the total positive area showed no differences between the groups (Fig. 2G, Fig. 2F, p = 0.75).

### K5 effects on vascular toxicity and SMCs content in neointima formation in vivo in the femoral artery cuff model

**Figure 3:**
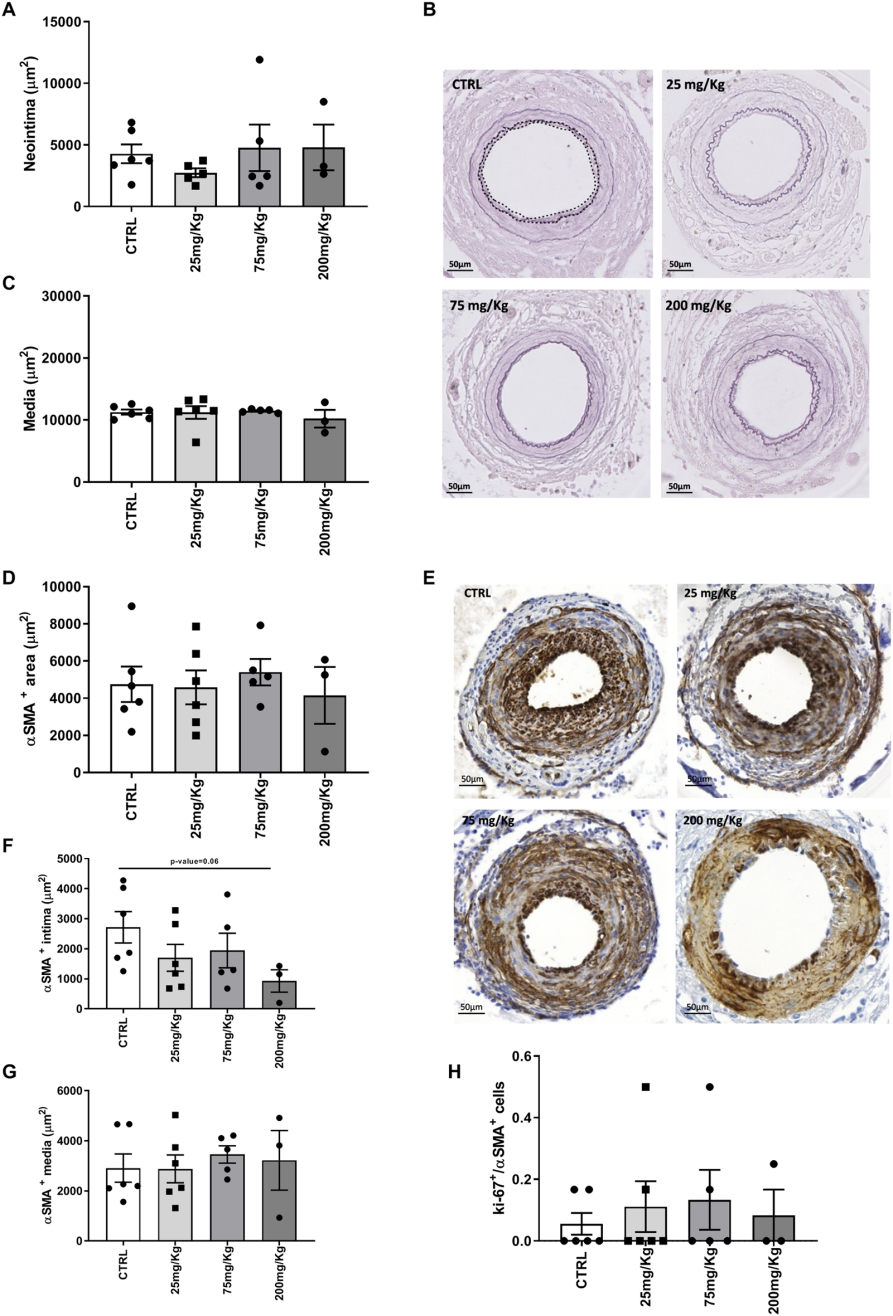
K5 does not affect SMCs driven neointima formation nor induce toxicity in femoral artery cuff lesions. (**A**) Quantification of the neointima area in control and K5 treated groups and (**B**) representative pictures of elastin staining of ctrl and K5 treated groups. The white dotted line delineates the perimeter of the lesion (**C**) Quantification of media area. (**D**) Quantification of αSMA positive area in the cuff-induced neointima model and (**E**) examples of pictures of αSMA IHC staining in control and K5 treated groups. (**F**) Quantification of αSMA positive area in intima and (**G**) media area of lesions from control and K5 treated mice. (**H**) Quantification of the number of proliferating smooth muscle cells in control and K5 treated groups. Data are presented as mean ± SEM. of n = 6 independent measurements per group *p < 0.05, **p < 0.01, ***p < 0.001. ****p < 0.0001; by 2-sided Student t test.

To determine whether K5 had an effect on SMCs driven neointima formation in vivo, we induced neointima formation in the femoral artery of C57BL/6 mice using a non-constrictive cuff and treated the mice with three different doses of K5 (25, 75 and 200 mg/kg). The lesions formed in these mice after cuff placement mainly consists of SMCs accumulating in the intimal layer (Fig. 3B, area between dotted lines).

Moreover, to determine whether K5 in different concentrations has toxic effects on the vessel wall, we studied the media size and breaks in the internal lamina as these parameters in this model are described as indicators of toxicity of the drug tested^27^. We did not observe differences in the medial area of mice treated with different concentrations of K5 and in the control group (Fig. 3C) nor could we detect differences in breaks in the internal lamina (examples of intact internal laminas in Fig. 3B). Taken together these parameters indicate that K5 used in these doses can be used safely and has no toxic side effects on the vessel wall.

We distinguished between lamina elastica externa and lamina elastica interna using an elastin immunohistochemical staining (Fig. 3B) and studied the neointima area. Comparable to the results obtained in the experiment with ApoE3*Leiden mice which underwent vein graft surgery, no differences could be detected in the neointima area of the treated groups when compared to the area of the control group, even with higher doses of K5 (Fig. 3A).

We found αSMA positive areas throughout the intimal and medial layers in all the K5 treated groups and in the control group (Fig. 3E). Quantification of the total area (media and intima) αSMA^+^ showed no differences in treated groups when compared to control (Fig. 3D). When looking at the two layers separately, we observed that SMCs content in the media of the control group was comparable to the content of the treated groups (Fig. 3G). The doses tested did not induce a significant reduction of intimal SMCs content when compared to the control group, although the highest dose tested of 200 mg/kg showed a trend toward a reduction in SMCs (*p* = 0.06) when compared to control (Fig. 3F). The number of proliferating SMCs was also comparable between the two groups (Fig. 3H) and no apoptotic SMC could be detected in the control group nor in the K5 treated groups (Supplementary Fig. 2).

### Effects of K5 on intraplaque angiogenesis and intraplaque hemorrhage in advanced atherosclerotic plaques

Since endothelial cells play a crucial role in the development and progression of atherosclerotic lesions we zoomed in on the effect of K5-mediated bFGF receptor binding blockade on endothelial cells in accelerated atherosclerotic vein graft lesions in ApoE3*Leiden mice.

**Figure 4:**
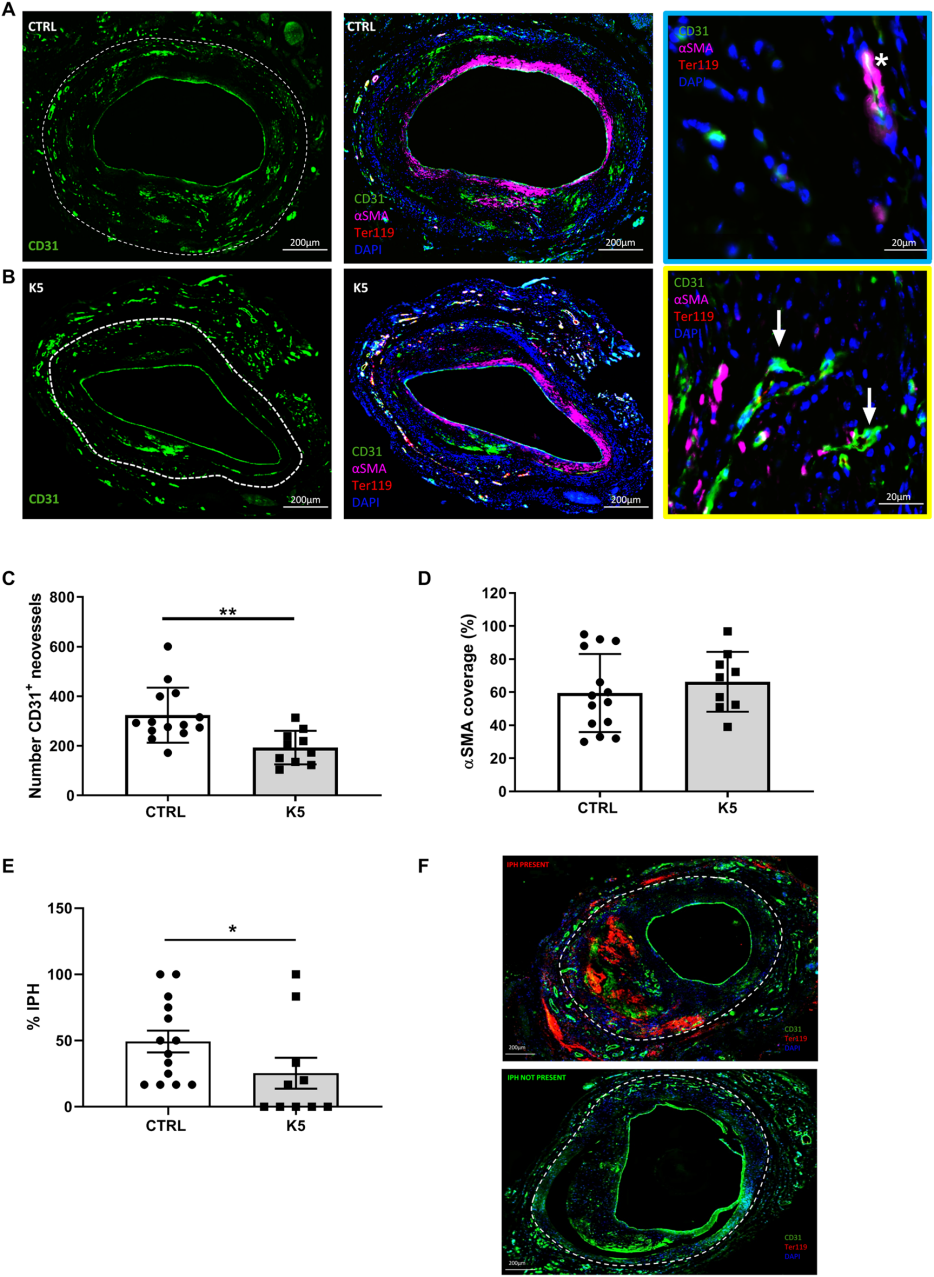
K5 impairs IP angiogenesis in advanced vein graft atherosclerotic plaques in ApoE3*Leiden mice. (**A**, **B**) Examples of atherosclerotic lesions stained for CD31 in the left panel, overview of the quadruple IHC staining for CD31, Ter119, αSMA and DAPI in the middle panel and example of mature (marked with a white star in the blue box) and immature vessels (marked with white arrows in the yellow box) in the right panel are shown (n = 14 in CTRL group and n = 10 in K5 group). (**C**) Quantification of IP angiogenesis in the control and K5 groups (n = 14 in CTRL group and n = 10 in K5 group). (**D**) Quantification of αSMA coverage (n = 14 in CTRL group and n = 10 in K5 group). (**E**) Quantification of IPH and (**F**) examples of IPH present (top panel) and IPH not present (bottom panel) (n = 14 in CTRL group and n = 10 in K5 group). Data are presented as mean ± SEM *p < 0.05, **p < 0.01, ***p < 0.001. ****p < 0.0001; by 2-sided Student t test.

An important aspect in this murine model of advanced atherosclerotic lesions is lumen re-endothelialisation. During the vein graft surgery procedure, the venous graft undergoes high distension due to high pressure of the arterial blood flow which results in loss of the endothelial cells at the luminal side. Restoration of the endothelial monolayer begins quickly after the initial damage and the endothelium surrounding the lumen is found to be intact four weeks after the surgery^28^. We found that the control mice had a complete lumen coverage, represented as a continuous ECs layer surrounding the lumen (Fig. 4A) and the same was visible also in the mice from the K5 treated group (Fig. 4B).

Endothelial cells not only are crucial for the coverage of the luminal side of the grafts, they also are the main cell type driving intraplaque angiogenesis. Therefore the effects of K5 on intraplaque angiogenesis was studied too. Thus we analysed the content of CD31^+^ neovessels in advanced atherosclerotic plaques in ApoE3*Leiden mice which underwent vein graft surgery and were treated with 25 mg/kg K5 (Fig. 4B) and compared it to the content of the control group (Fig. 4A). CD31 positive neovessels were found throughout the vessel wall area of both control and treated mice (area outlined with white dotted lines in Fig. 4A and B), but interestingly K5 treated mice showed reduced numbers of neovessels when compared to the control group (*p* = 0.003, Fig. 4C).

Next, we evaluated the maturity of the neovessels and quantified the percentage of vessels that presented a pericyte coverage on top of the ECs layer. Mature vessels present an EC layer fully covered by a SMCs layer (example in Fig. 4, zoom-in box outlined in blue) and immature vessels present only an ECs layer (example in Fig. 4, zoom-in box outlined in yellow). No significant difference in the amount of SMCs coverage was found when comparing mice of the control group to the mice of the K5 group (Fig. 4D, p value = 0.4).

A complication of immature neovessels is the extravasation of red blood cells, phenomenon known as intraplaque haemorrhage (IPH). We determined the degree of intraplaque haemorrhage in the lesions of control and K5 treated mice and scored them as present (Fig. 4F, top panel) or not present (Fig. 4F, bottom panel). As shown in the quantification in Fig. 4E, we observed a reduction in the amount of IPH in the lesions of mice treated with K5 when compared to controls (*p* = 0.04).

### K5 reduces circulating monocytes and macrophage content in accelerated atherosclerotic lesions

**Figure 5:**
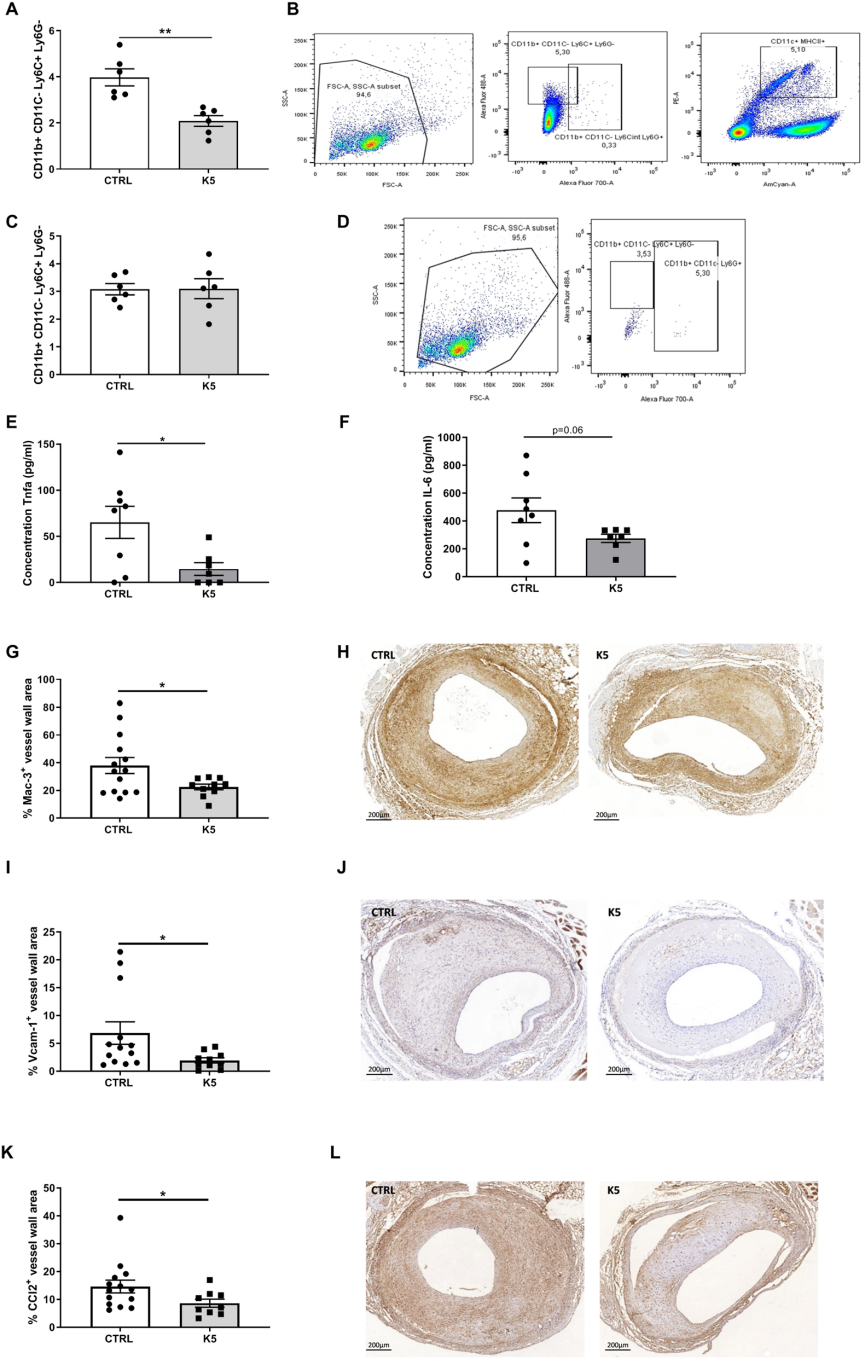
K5 reduces circulating monocytes and macrophage infiltration in advanced vein graft atherosclerotic lesions in ApoE3*Leiden mice. (**A**) FACS quantification of circulating monocytes in the blood of control and K5 treated mice and (**B**) gating strategy (n = 6 mice per group). (**C**) FACS quantification of monocytes in the spleen of control and K5 treated mice and (**D**) gating strategy (n = 6 mice per group). Quantification of the concentration of Tnf (**E**) and IL-6 (**F**) in samples from whole blood assay incubated with LPS (n = 8 mice per group). (**G**) Quantification of Mac3 positive area in the control and K5 groups and (**H**) respective example of the staining on the right (n = 14 in CTRL group and n = 10 in K5 group). (**I**) Quantification of Vcam-1 and (K) Ccl-2 positive area in the control and K5 groups (n = 14 in CTRL group and n = 10 in K5 group). (**J**) Representative pictures for Vcam-1 IHC staining and (**L**) Ccl2 IHC staining in mice from ctrl and K5 groups (n = 14 in CTRL group and n = 10 in K5 group). Data are presented as mean ± SEM. *p < 0.05, **p < 0.01, ***p < 0.001. ****p < 0.0001; by 2-sided Student t test.

bFGF was previously shown to be a mitogen for multipotent progenitors from bone marrow, mostly in the myeloid lineage^29–31^. Although ineffective by itself, it is thought to potentiate the effects of other growth factors and thus act as a permissive factor^32^. Therefore, we assessed the effect of bFGF blockage on systemic inflammation, examining the number of circulating monocytes. CD11b^+^, CD11C^-^, Ly6C^+^, Ly6G^-^ cells were found to be present in both control and K5 treated mice in the blood and spleen (Fig. 5B and D respectively). Quantification of the number of cells showed that circulating monocytes were significantly reduced in the K5 treated group when compared to control (Fig. 5A, p = 0.001) while no differences were detected between groups when quantifying the amount of monocytes in the spleen (Fig. 5C).

To examine the activity of the available cells we examined the amount of pro-inflammatory cytokines in the blood of mice from the control and K5 treated groups. One of the main functions of monocytes is to secrete cytokines so we looked at the amount of pro-inflammatory cytokines after 24 h treatment with LPS in vitro. TNF-alpha production in whole blood in mice treated with K5 was significantly reduced when compared to whole blood from mice of the control group (Fig. 5E p = 0.02). The production of IL-6 also decreased in whole blood from mice of the K5 group when compared to the control group upon stimulation with LPS (Fig. 5F. *p* = 0.06).

To analyse the consequences of lower numbers of circulating monocytes on plaque composition and inflammation, we studied the effect of K5 on intraplaque inflammation in the vein graft lesions. Mac3 Positive cells were found throughout the lesion in both control and K5 treated group (Fig. 5H), but in the lesions of K5 treated mice macrophages were significantly reduced by 15% when compared to control (Fig. 5G, p = 0.03).

Since it is known that bFGF upregulates the expression of the cell adhesion molecule VCAM-1 in ECs, increasing polymorphonuclear leukocyte adhesion and trans-endothelial migration^33^ and moreover, it can also upregulate the expression of a number of chemokines involved in the recruitment of different inflammatory cells like CCL2, we investigated whether the decrease in macrophages could also be due to reduced infiltration of monocytes in the lesions, by reduction of VCAM-1 expression or reduced CCL2 expression.

VCAM-1 was found to be expressed in the lumen endothelium, as well as in the neovessels in the medial and intimal layers of the vessel wall (Fig. 5J). CCL2 was present throughout the vessel wall in both control and treated groups (Fig. 5L). Interestingly both VCAM- 1 and CCL2 were found to be significantly decreased, by 5 and 6% respectively, in the lesions of K5 treated mice when compared to control (Fig. 5I and K).

### Effect of K5 on the number of neovessels in an in vivo Matrigel plug model

Since the effects on intraplaque angiogenesis in the vein graft model may depend on direct and indirect factors including the size of the lesions, inflammation and degree of ischemia in those lesions, we evaluated the effects of K5 on angiogenesis directly. For this we used an in vivo Matrigel plug model in C57BL/6, in which we tested the effect of K5 in multiple doses (25, 75 and 200 mg/Kg) on angiogenic vessel influx in the plugs.

**Figure 6:**
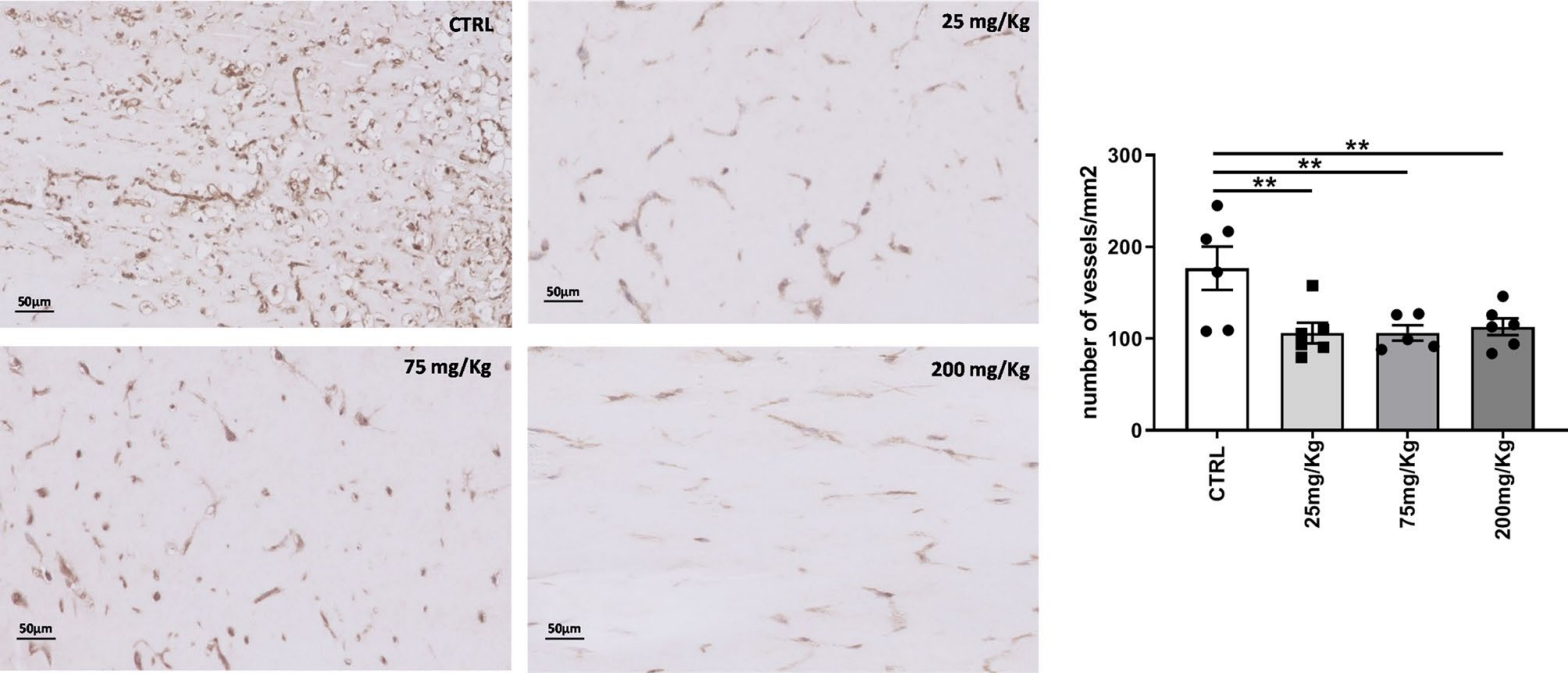
K5 reduces the number of vessels formed in a Matrigel plug model. Examples of CD31 positive neovessels in Matrigel plugs from control mice and mice treated with different increasing doses of K5. On the right quantification of the number of neovessels in the control and treated groups. Data are presented as mean ± SEM. of n = 6 independent measurements per group *p < 0.05, **p < 0.01, ***p < 0.001. ****p < 0.0001; by 2-sided Student t test.

Mice treated with K5 showed a reduction in CD31^+^ neovessels when compared to control (shown in Fig. 6). Quantification of the staining confirmed the difference observed and resulted in a significant reduction of CD31 positive vessels in all the treated group when compared to control (*p* value = 0.008).

Differently from the results obtained in the vein graft model in ApoE3*Leiden mice, no differences could be found in the expression of VCAM-1 and CCL-2 in the Matrigel plugs of the mice treated with K5 when compared to control (Supplementary Figs. 3 and 4 respectively), while we observed a significant reduction in the number of macrophages in the group treated with the highest dose of K5 (200 mg/Kg) when compared to control (Supplementary Fig. 3, *p* = 0.02).

### In vitro angiogenesis is reduced upon K5 treatment

To understand how K5 impairs ECs functions, we investigated its effect in different ECs functional assays in vitro. Namely MTT assay to study ECs proliferation, scratch wound healing assay to evaluate ECs migration, and tube formation assay to assess the ability of ECs to form tubular structures that resemble new vessels.

**Figure 7:**
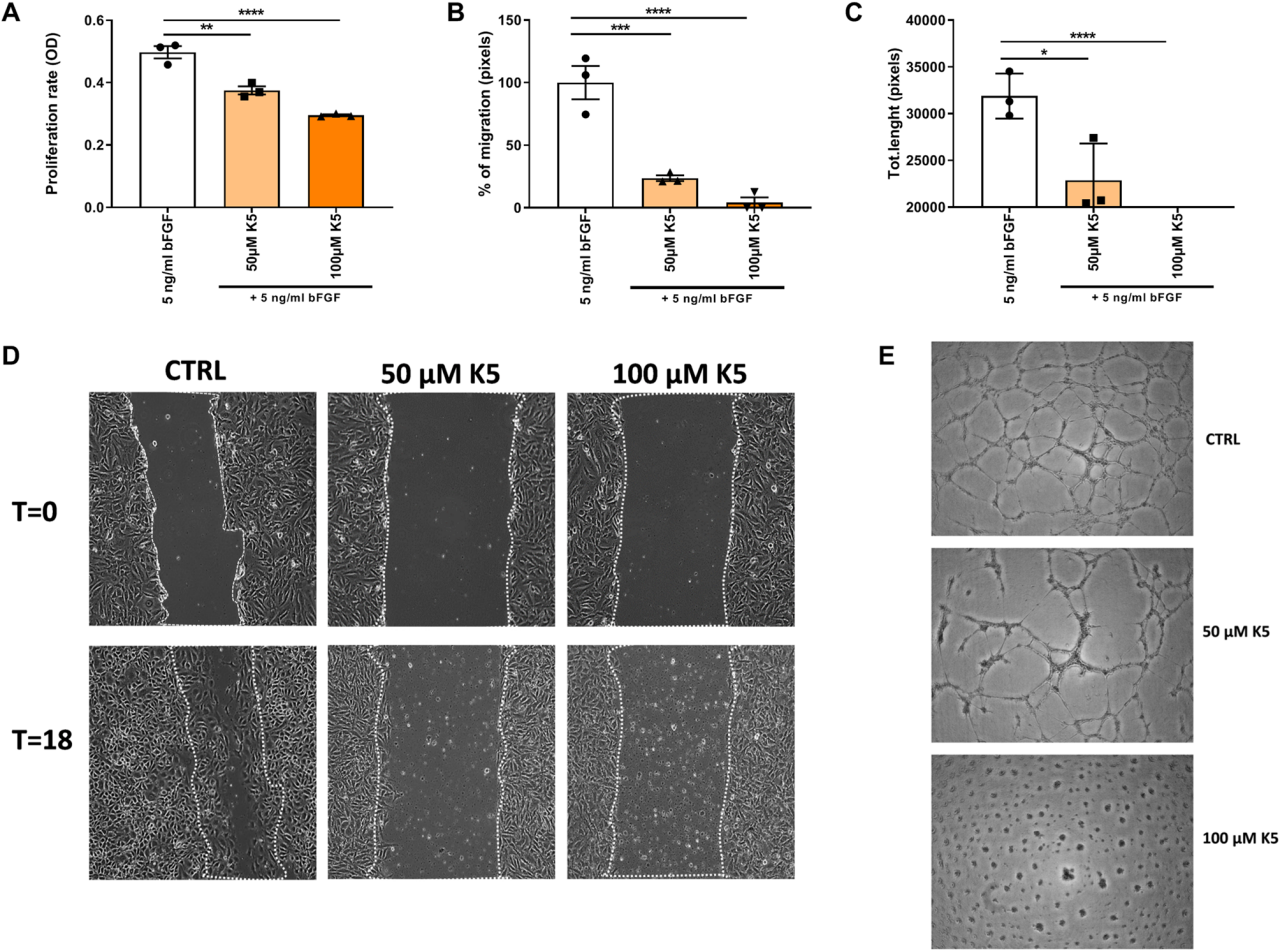
K5 impairs in vitro angiogenesis. (**A**) Quantification of proliferation rate of H5V cells treated with K5 compared to control (n = 3 technical replicates). (**B**) Quantification of migration rate of H5V cells at T18 (n = 3 technical replicates) and (**D**) representative pictures of wound healing scratches at T0 and after 18 h. (**C**) Quantification of total length of tube formed after 12 h of control and K5 treated groups (n = 3 technical replicates). (**E**) Examples of HUVECs tube formation in control and K5 treated groups and quantification method. Data are presented as mean ± SEM. *p < 0.05, **p < 0.01, ***p < 0.001. ****p < 0.0001; by one way ANOVA.

K5 significantly reduced H5V proliferation rate when compared to control as shown in Fig. 7A. In fact ECs proliferation was reduced by 24.5% and 40.6% in the cells treated with 50 µM and 100 µM K5 respectively (*p* = 0.0014 and < 0.0001).

We next performed a scratch wound healing assay in H5V cells and treated the cells with increasing doses of K5. Treatment with K5 significantly decreased scratch wound closure when compared to control by 76.3% and 95.8% in the groups treated with 50 and 100 µM respectively (Fig. 7B and D). Quantification of the migration rate showed a dose dependent significant reduction in ECs migration ability with all the doses of K5 tested when compared to control (Fig. 7B and D, p = 0.0002 and < 0.0001 respectively).

We further looked at the ability of K5 to impair the capability of ECs to form capillary like structures and evaluated the total length of tubes formed (Fig. 7C and E). K5 resulted to be able to strongly reduce HUVECs tube formation by 14% with the lowest dose tested of 50 µM (*p* = 0.01) and to completely inhibit tube formation with the dose of 100 µM (Fig. 7C and E, p =  < 0.0001) reducing the formation of tubes by 100%.

## Discussion

In the present study we found that K5 mediated inhibition of bFGF reduces intraplaque angiogenesis and intraplaque haemorrhage in a model for accelerated atherosclerosis using vein graft surgery in ApoE3*Leiden mice. Additionally, K5 reduced in vivo angiogenesis in a Matrigel plug model. We demonstrate that K5 is able to impair EC migration, proliferation and tube formation due to a reduced FGFR1 activation in vitro. Moreover, we found that K5 is able to reduce macrophage infiltration in the plaque via modulating the expression of VCAM-1 and CCL-2.

bFGF is known to play a crucial role during angiogenesis. bFGF was shown to be present in complicated atherosclerotic plaques and in higher amounts in patients who presented symptomatic carotid disease when compared to stable plaques^5^. High expression of bFGF/FGFR-1 was also associated with immature and inflammatory intraplaque angiogenesis and plaque instability in a rabbit model of atherosclerosis^13^. In this study we demonstrate that the small molecule K5 can block bFGF signalling, which leads to a decrease in in vitro angiogenesis via inhibition of ECs migration, proliferation and tube formation due to a reduction of FGFR1 phosphorylation. The above mentioned features lead to a decrease of in vivo angiogenesis in a Matrigel plug model and more importantly a reduction of intraplaque angiogenesis in accelerated atherosclerotic lesions in vein grafts in hypercholesterolemic ApoE3*Leiden mice. Our findings are in line with a study by Tanaka et al., in which FGF-2 treatment increased the number of vasa vasorum in atherosclerotic lesion formation^34^. Moreover FGF-2/FGFR-1 signalling was found to be critical for providing a pattern for the vasa vasorum to form a plexus-like network of neovessels in the atherosclerotic lesions in hypercholesterolemic low-density lipoprotein receptor–deficient/apolipoprotein B^100^^/100^ mice^35^.

A mature neovessel is formed by a basement membrane, a layer of ECs and pericyte coverage. The amount of intraplaque haemorrhage decreased in the treated group when compared to the control. It was suggested that exposure to bFGF hampers endothelial cell–cell junctions^36^. This could explain why bFGF blockade via K5 lead to reduction in intraplaque haemorrhage, probably via enhancing cell–cell connections between endothelial cells.

In 1991 Reidy & Lindner showed that an antibody raised against bFGF could reduce the proliferation of SMCs in a rat model in which carotid arteries where denuded of endothelium with a balloon catheter^20^. Interestingly, in their study this treatment did not lead to any significant decrease in the intimal lesion. Although in the present study, in both the murine model for accelerated atherosclerosis and in the model for SMCs driven neointima formation, we could not observe any difference in SMCs proliferation in the treated groups compared to control, we show that the blockade of bFGF signalling by K5 did not affect intimal lesion area similarly to what observed by Reidy et al. More recently Chen et al., showed that blockage of the signalling of all four FGFRs (FGFR1, 2, 3 and 4) through inhibition of Frs2α, a fibroblast growth factor receptor substrate, resulted in decrease SMCs content and reduced lesion size in ApoE^-/-^ mice^37^. In our study we demonstrate that small molecule mediated blockade of only bFGF does not affect the lesion’s SMCs content while still reducing intraplaque angiogenesis.

In the present study we showed that K5-mediated bFGF signalling blockade induces a reduction in systemic levels of monocytes. This is in accordance with previous studies, which demonstrated that bFGF acts as a mitogen for multipotent progenitors from bone marrow, mostly in the myeloid lineage^29–31^. Moreover, we demonstrate that K5 reduced the amount of macrophages present in the lesions via reducing the expression of VCAM-1 and CCL-2, both proteins correlated with macrophage infiltration. Similarly, Liang et al., 2018 showed that knockout of FGF2 reduced infiltration and accumulation of macrophages at different stages of atherosclerosis. They also showed that the knockout induced a reduction in CCL-2 and VCAM-1 expression in the atherosclerotic plaques^6^. Therefore the observed reduced macrophages content in the ApoE3*Leiden mice treated with K5 could be either due to the reduced number of circulating monocytes or a reduced monocyte infiltration in the lesion probably due to a reduced CCL-2 and VCAM-1 expression or a combination of both processes. However the effect of K5 on intraplaque angiogenesis and its effect on the expression of CCL-2 and VCAM-1 could be unrelated to its effect on monocytes and macrophages.

The accelerated atherosclerosis vein graft model used in the present study is a unique model to study plaque angiogenesis. Intraplaque angiogenesis is a feature that is uncommon in murine models of native atherosclerosis except for very old mice. The lesions observed in the ApoE3*Leiden mice vein grafts show many features that can also be observed in advanced human lesions, including intraplaque hypoxia, angiogenesis and intraplaque haemorrhage. However this is a model in which a vein is inter-positioned in the arterial circulation which makes it an acute model, with high grade inflammation and extensive remodelling. Extrapolation of the effects of K5 in this model to native atherosclerosis should be carefully performed with taking the previous mentioned processes into account. Based on the results obtained in the present study we can conclude that K5 is able to stabilize the atherosclerotic plaque by enhancing plaque stability via reducing intraplaque angiogenesis and decreasing intraplaque haemorrhage. Moreover, it reduces systemic circulating monocytes and decreases macrophages infiltration in the plaque. Taken together, our results show that K5 is a promising therapeutic candidate for the treatment of unstable atherosclerotic plaques.

## Materials and methods

### Mice

This study was performed in compliance with Dutch government guidelines and the Directive 2010/63/EU of the European Parliament. All animal experiments were approved by the animal welfare committee of the Leiden University Medical Center.

For vein graft surgery male ApoE3*Leiden mice (n = 14 for the control group and n = 10 for the K5 treated group), crossbred in our own colony on a C57BL/background, 8–16 weeks old, were fed a diet containing 15% cacao butter, 1% cholesterol and 0.5% cholate (100,193, Triple A Trading, Tiel, The Netherlands) from 3 weeks prior to surgery until sacrifice. Mice were randomized based on their plasma cholesterol levels (kit 1,489,437, Roche Diagnostics, Basel, Switzerland) and body weight.

For femoral artery cuff model (n = 3 per group) and Matrigel plug model (n = 3 per group) 8 weeks old male C57BL/6 J mice were used and were fed a chow diet. Mice were randomized in four different groups.

### Anesthesia

For all the above-mentioned surgical procedures, on the day of surgery and on the day of sacrifice mice were anesthetized as previously described^25,38^. Briefly, mice were anesthetized with midazolam (5 mg/kg, Roche Diagnostics, Basel, Switzerland), medetomidine (0.5 mg/kg, Orion, Espoo, Finland) and fentanyl (0.05 mg/kg, Janssen Pharmaceutical, Beerse, Belgium). The adequacy of the anesthesia was monitored by keeping track of the breathing frequency and the response to toe pinching of the mice. After surgery, mice were antagonized with atipamezol (2.5 mg/kg, Orion, Espoo, Finland) and fluminasenil (0.5 mg/kg, Fresenius Kabi, Bad Homburg, Germany). Buprenorphine (0.1 mg/kg, MSD Animal Health, Keniworth, NJ, USA) was given after surgery to relieve pain. On the day of sacrifice mice underwent deep anesthesia with midazolam (5 mg/kg, Roche Diagnostics, Basel, Switzerland), medetomidine (0.5 mg/kg, Orion, Espoo, Finland) and fentanyl (0.05 mg/kg, Janssen Pharmaceutical, Beerse, Belgium) and were then euthanized by exsanguination.

### Vein graft surgery

Vein graft surgery was performed by donor mice caval vein interposition in the carotid artery of recipient mice as previously described^25^. Briefly, thoracic caval veins from donor mice were harvested. The right carotid artery of the recipient mouse was dissected and cut in the middle. The artery was everted around the cuffs that were placed at both ends of the artery and ligated with 8.0 sutures. The caval vein was sleeved over the two cuffs, and ligated. After 28 days, mice were anesthetized and sacrificed via perfusion with 4% formaldehyde. Vein grafts were harvested, fixed in formaldehyde and paraffin-embedded.

### Femoral artery cuff mouse model

C57BL/6 mice underwent a non-constrictive cuff placement around the femoral artery to induce vascular remodeling as previously described^39^. Briefly, the left and right femoral arteries were isolated and a rigid, non-constrictive polyethylene cuff was placed around the artery. Thereafter, the wound was closed by a continuous suture. After 21 days, mice were anesthetized and sacrificed via perfusion with 4% formaldehyde. Cuffed femoral arteries were harvested, fixed in formaldehyde and paraffin-embedded.

### Matrigel plugs

In vivo angiogenesis analysis was performed using a Matrigel plug assay in male C57BL/6 mice as previously described^40,41^. Matrigel extracellular matrix (Ref. 354,262, Corning, NY, USA) was mixed at 4 °C with PBS and supplemented with 50 ng/ml bFGF (Ref. 579,606, Biolegend, California, USA). The solution was then injected into the subcutaneous space on the dorsal side of mice on both the left and right flank (350 µl per flank). Mice were sacrificed 21 days post-implantation. Matrigel plugs were excised, fixed in formaldehyde, paraffin-embedded and processed for histological analysis.

### K5 synthesis and application

The bFGF signalling blockade inhibitor K5 was synthesized according to the procedure described in Example 10 of US Patent 9,181,196^24^. ^1^HNMR, ^13^CNMR, and FT-IR spectroscopies were adopted to ascertain correspondence of the synthesized compound employed in this study to 3,3′-(propane-1,3-diyilbis(azanediyl)bis(oxomethylene)bis(1-(2,4-dichlorophenyl)-1,4-dihydro-thieno[3′,2′:4,5]cyclohepta[1,2-c]pyrazole-8-sulfonic acid), namely K5 (Fig. 1).

K5 was dissolved in water for injections (Fresenius, Kabi) at a final concentration of either 25, 75 or 200 mg/kg. For the accelerated atherosclerosis vein graft model mice were treated with IP injections of 25 mg/kg K5 or vehicle (water for injections, Fresenius Kabi) every other day starting at day 14 until the sacrifice on day 28. For the cuff model and the Matrigel plug model mice were treated with IP injections of either 25, 75 or 200 mg/kg K5 or vehicle (water for injections, Fresenius Kabi) every other day starting at day 3 until the sacrifice on day 21.

### Histological and immunohistochemical assessment of vein grafts, cuffs and Matrigel plugs

Histological and immunohistochemical assessment of vein grafts, cuffs and Matrigel plugs was performed as previously described^25,38^. In detail, vein grafts, cuffs and Matrigel plugs histological samples were embedded in paraffin, and sequential cross-Sects. (5 μm thick) were made throughout the specimens. For each mouse, six equal spaced cross-sections over the total vein graft, cuffs and Matrigel plugs length were used for analysis. To quantify the vein graft thickening (vessel wall area), MOVAT pentachrome staining was performed. Total size of the vein graft and lumen were measured. Thickening of the vessel wall (measured as intimal thickening + media thickening) was defined as the area between lumen and adventitia and determined by subtracting the luminal area from the total vessel area. Intraplaque angiogenesis was measured as the amount of CD31^+^ vessels in the vessel wall area and the percentage of neovessels CD31 + αSMA + was defined as % αSMA coverage. Intraplaque hemorrhage (IPH) was monitored by the amount of erythrocytes outside the (neo)vessels and scored as either present or not present. Antibodies directed at alpha smooth muscle cell actin (αSMActin, Sigma, Santa Clara, CA, USA), Mac-3 (BD Pharmingen, Franklin Lakes, NJ, USA), CD31 (77699S, Cell Signaling, Danvers, MA, USA), Ter119 (116,202, Biolegend, San Diego, CA, USA), Ki67 (ab16667, Abcam, Cambridge, UK), MCP-1 (sc-1784, Santa Cruz Biotechnology, Dallas, TX, USA), VCAM-1 (ab134047, Abcam, Cambridge, UK) and cleaved caspase 3 (9661-S, Cell SignalingDanvers, MA, USA) were used for immunohistochemical staining. Sirius red staining (80,115, Klinipath, Amsterdam, The Netherlands) was performed to quantify the amount of collagen present in the vein grafts. The immuno-positive areas are expressed as a percentage of the lesion area.

Paraffin sections of femoral arteries from the cuff model experiment were stained with Weigert’s Elastin to visualize the elastic laminae to determine intimal hyperplasia. Smooth muscle cells were stained using anti-alpha smooth muscle cell actin (αSMActin, Sigma, Santa Clara, CA, USA) and their proliferation was examined using anti-Ki-67 (ab16667, Abcam, Cambridge, UK) antibody.

Matrigel plugs paraffin sections were stained using anti-CD31 antibody (sc-1506-r, Santa Cruz Biotechnology, Dallas, TX, USA) to evaluate the number of CD31^+^ neovessels.

Stained slides were photographed using Pannoramic MIDI II digital slide scanner (3DHISTECH, Budapest, Hungary) and image analysis softwares were used to quantify the vein graft intimal hyperplasia and composition (Qwin, Leica, Wetzlar, Germany and Imagej, Bethesda, MD, USA).

### Flow cytometry

Flow cytometry was performed on spleen and blood of n = 6 mice per group 28 days after vein graft surgery. Single cells suspensions were prepared from spleens by mincing the tissue through a 70 µm cell strainer (BD Biosciences, San Jose, CA, USA). Cells were washed with 10 ml IMDM Glutamax (ThermoFisher, Waltham, Massachusetts, USA) with 8% heat inactivated fetal bovine serum (PAA, Australia) and 100 U/mL Penicillin/streptomycin. Erythrocytes were lysed in red blood cell lysis buffer (hypotonic ammonium chloride buffer). Conjugated monoclonal antibodies to mouse CD11b (V450), Ly6C (FITC/Alexa488), Ly6G (Alexa Fluor 700), CD11c (PE), were purchased from eBioscience or BD Biosciences. Dead cells were excluded by positivity for 7-aminoactinomycinD (7-AAD) (Invitrogen, ThermoFisher, Waltham, Massachusetts, USA). Flow cytometric acquisition was performed on a BD LSR II flow cytometer (BD Biosciences). Data were analyzed using FlowJo V10.1 software.

### Whole blood assay and determination of cytokine production

Blood was collected from mice of the control group (n = 4) and of the K5 treated group (n = 4) on the day of sacrifice 28 days after vein graft surgery. Blood was diluted 25X in either RPMI 1640 medium (52,400–025, ThermoFisher, GIBCO, Waltham, Massachusetts, USA) or RPMI 1640 medium supplemented with 200 ng/ml LPS (K-235, Sigma-Aldrich, Saint Louis, Missouri, USA) and incubated at 37 °C in a humidified 5% CO_2_ environment for 24 h. The supernatant was then collected and used to perform ELISA for cytokine concentration of TNFa and IL 6 using ELISA kit 558,534 (BD Bioscience) and ELISA kit 555,240 (BD Bioscience) respectively.

### Cell culture

For the isolation of HUVECS anonymous umbilical cords were obtained in accordance with guidelines set out by the ‘Code for Proper Secondary Use of Human Tissue’ of the Dutch Federation of Biomedical Scientific Societies (Federa), and conform to the principles outlined in the Declaration of Helsinki. Isolation and culturing of primary vein human umbilical cells was performed as described by Van der Kwast et al.^42^. HUVECs were cultured in plates coated with 1% fibronectin. Cells were used between passage 2 and 4.

H5V (endothelial cell line derived from murine heart) cells were kindly provided by Dr. I. Bot from the Leiden Academic Centre for Drug Research.

H5V and HUVECs were cultured at 37 °C in a humidified 5% CO_2_ environment. Culture medium was refreshed every 2 days. Cells were passed using trypsin–EDTA (Sigma, Steinheim, Germany) at 90–100% confluency.

### Detection of bFGF in culture medium

bFGF concentrations in the culture medium of H5V cells treated with 5 ng/ml bFGF (Ref.579606, Biolegend) or 5 ng/ml bFGF plus either 50 or 100 µm K5, were determined using the Mouse FGF basic/FGF2 DuoSet ELISA Kit (DY3139-05, R&D Systems, Minneapolis, MN, USA), according to the manufacturer’s instructions.

### FGFR1 phosphorylation assay

HUVECs were seeded in 12-well plates with EBM-2 Basal medium (CC-3156, Lonza) supplemented with EGM-2 SingleQuots Supplements (CC-4176, Lonza) for 24 h. Cells were then stimulated with 5 ng/ml bFGF (Biolegend) or 5 ng/ml bFGF in combination with either 50 or 100 µm K5 for 60 min. The cells were rinsed with PBS and lysed in cell lysis buffer containing IC diluent #12 (Reagent diluent concentrate 2 DY995, R&D Systems, Minneapolis, MN, USA, in distilled water), plus 10 µg/ml aprotinin (Ref.4139, R&D Systems, Minneapolis, MN, USA) and 10 µg/ml leupeptin (Ref.1167, R&D Systems, Minneapolis, MN, USA). FGFR1 phosphorylation was measured by a sandwich ELISA (DYC5079-2, R&D Systems, Minneapolis, MN, USA) according to the manufacturer’s instructions.

### Cell proliferation

Cell proliferation (n = 3 individual replicates) was measured using MTT assay. H5V cells were plated at 5.000 cells/well in a 96 well plate and grown until 80–85% confluency in complete culture medium (DMEM GlutaMAX (Invitrogen, GIBCO, Auckland, New Zealand), 10% heat inactivated fetal bovine serum (PAA), 1% penicillin/streptomycin, after which they were incubated with low serum medium (DMEM GlutaMAX (Invitrogen, GIBCO, Auckland, New Zealand), 0.1% heat inactivated fetal bovine serum (PAA), 1% penicillin/streptomycin) for 24 h. The medium was then replaced by treatment mixes consisting of low serum medium plus 5 ng/ml bFGF (Ref.579606, Biolegend), low serum medium plus 5 ng/ml bFGF (Ref.579606, Biolegend) with the addition of either 50 or 100 µM K5. After 24 h incubation, 10µL MTT (Thiazolyl blue tetrazolium bromide, Sigma M5655) was added directly to each well and cells were incubated at 37 °C in a humidified 5% CO_2_ environment for 4 h. Subsequently, 75µL medium was removed from each well and 75µL isopropanol/0.1 N HCL was added per well. After incubating the plate for 90 min on a shaker platform, absorbance was read at 570 nm with a Cytation 5 spectrophotometer (BioTek, Vermont, USA).

### Scratch wound healing assay

For the scratch wound healing assay (n = 3 individual replicates), H5V cells were plated on a 12 well plate and grown until 80% confluence in complete culture medium (DMEM GlutaMAX (Invitrogen, GIBCO, Auckland, New Zealand), 10% heat inactivated fetal bovine serum (PAA), 1% penicillin/streptomycin). Cells were then treated with low serum medium (DMEM GlutaMAX (Invitrogen, GIBCO, Auckland, New Zealand), 0.1% heat inactivated fetal bovine serum (PAA), 1% penicillin/streptomycin) for 24 h. After 24 h, medium was removed and a scratch-wound was introduced across the diameter of each well of a 12 wells plate using a p200 pipette tip. Subsequently, the cells were washed with PBS and medium was replaced by new low serum culture medium containing low serum medium plus 5 ng/ml bFGF (Ref.579606, Biolegend), and low serum medium plus 5 ng/ml bFGF (Ref.579606, Biolegend) with the addition of either 50 or 100 µM K5. Two locations along the scratch-wound were marked per well and scratch-wound closure at these sites was imaged by taking pictures at time 0 h and 18 h after scratch-wound introduction using live phase contrast microscopy (Axiovert 40C, Carl Zeiss Microscopy, White Plains, NY, USA). Average scratch-wound closure after 18 h was objectively calculated per well by measuring difference in cell coverage at 18 h vs 0 h using the wound healing tool macro for ImageJ.

### Tube formation assay

HUVECs were seeded in 12-well plates with EBM-2 Basal medium (CC-3156, Lonza) supplemented with EGM-2 SingleQuots Supplements (CC-4176, Lonza) until confluent. Medium was then replaced with low serum culture medium EBM-2 Basal medium (CC-3156, Lonza) supplemented with 0.2% FCS and 1% GA-1000 (EGM-2 SingleQuots Supplements, CC-4176, Lonza) for 24 h. A 96-wells was coated using 50 µl/well of Geltrex extracellular matrix (A1413202, Gibco). Cells were then detached using trypsin–EDTA (Sigma, Steinheim, Germany) and diluted at 150.000 cells/ml in low serum medium plus 5 ng/ml bFGF (Ref.579606, Biolegend), or low serum medium plus 5 ng/ml bFGF (Ref.579606, Biolegend) with the addition of either 50 or 100 µM K5. After 12 h incubation pictures of each well were taken using live phase contrast microscopy (Axiovert 40C, Carl Zeiss). Total length of the tubes formed was analyzed using the Angiogenesis analyzer plugin for ImageJ**.**

### Statistical analysis

Results are expressed as mean ± SEM. A 2-tailed Student’s t-test or a One-Way ANOVA were used to compare individual groups. Non-Gaussian distributed data were analyzed using a Mann–Whitney U test using GraphPad Prism version 6.00 for Windows (GraphPad Software). Probability-values < 0.05 were regarded significant.

## Supplementary information

Supplementary Information.

## Data Availability

All data generated or analysed during this study are included in this published article (and its Supplementary Information files).
