# Supplementary material for: bFGF blockade reduces intraplaque angiogenesis and macrophage infiltration in atherosclerotic vein graft lesions in ApoE3*Leiden mice
Source: Sci Rep. 2020 Sep 29;10:15968. doi: 10.1038/s41598-020-72992-7 (PMC7525538; doi:10.1038/s41598-020-72992-7)
Supplement: Supplementary file 1 — Supplementary Information. [file 41598_2020_72992_MOESM1_ESM.docx]

**SUPPLEMENTARY INFORMATION**

**bFGF blockade reduces intraplaque angiogenesis and macrophage infiltration in atherosclerotic vein graft lesions in ApoE3*Leiden mice**

Laura Parma^1,2^, Hendrika A. B. Peters^1,2^, Thijs J. Sluiter^1,2^, Karin H. Simons^1,2^, Paolo Lazzari^3^, Margreet R. de Vries^1,2^ and Paul H.A. Quax^1,2^

**Correspondence should be addressed to P.H.A. Quax** (P.H.A.Quax@lumc.nl)

P.H.A Quax

Department of Vascular Surgery, D6-33

Leiden University Medical Center

PO Box 9600, 2300 RC Leiden, The Netherlands

Tel: +31 71 526 1584; Fax: +31 71 526 6750; Email: [P.H.A.Quax@lumc.nl](mailto:P.H.A.Quax@lumc.nl)

**Supplementary figure 1.** Bodyweight and cholesterol levels. (A). Bodyweight before (t=0) and 28 days after surgery (t=28) of control and K5 treated mice. (B). Plasma cholesterol levels before (t=0) and 28 days after surgery (t=28) of control and K5 treated mice.

**Supplementary information 2.** Representative pictures of femoral artery cuffs of ctrl and K5 treated groups stained for DAPI (blue), αSMA (green) and Cleaved caspase 3 (magenta).

**Supplementary information 3.** Representative pictures of Matrigel plugs of ctrl and K5 treated groups stained for DAPI (cyan), Mac3 (magenta) and VCAM-1 (yellow) and respective quantifications. Data are presented as mean±SEM *p<0.05; by one way Anova.

**Supplementary information 4.** Representative pictures of Matrigel plugs of ctrl and K5 treated groups stained for DAPI (cyan) and CCL-2 (red) and respective quantification. No significant differences could be observed by one way Anova.
